# Multigroup prediction in lung cancer patients and comparative controls using signature of volatile organic compounds in breath samples

**DOI:** 10.1371/journal.pone.0277431

**Published:** 2022-11-30

**Authors:** Shesh N. Rai, Samarendra Das, Jianmin Pan, Dwijesh C. Mishra, Xiao-An Fu

**Affiliations:** 1 Biostatistics and Bioinformatics Facility, Brown Cancer Center, University of Louisville, Louisville, KY, United States of America; 2 School of Interdisciplinary and Graduate Studies, University of Louisville, Louisville, KY, United States of America; 3 Hepatobiology and Toxicology Center, University of Louisville, Louisville, KY, United States of America; 4 Department of Bioinformatics and Biostatistics, University of Louisville, Louisville, KY, United States of America; 5 Biostatistics and Informatics Facility, Center for Integrative Environmental Research Sciences, University of Louisville, Louisville, KY, United States of America; 6 Christina Lee Brown Envirome Institute, University of Louisville, Louisville, KY, United States of America; 7 ICAR-Directorate of Foot and Mouth Disease, Arugul, Bhubaneswar, Odisha, India; 8 International Centre for Foot and Mouth Disease, Arugul, Bhubaneswar, Odisha, India; 9 ICAR-Indian Agricultural Statistics Research Institute, PUSA, New Delhi, India; 10 Department of Chemical Engineering, University of Louisville, Louisville, KY, United States of America; Cranfield University, UNITED KINGDOM

## Abstract

Early detection of lung cancer is a crucial factor for increasing its survival rates among the detected patients. The presence of carbonyl volatile organic compounds (VOCs) in exhaled breath can play a vital role in early detection of lung cancer. Identifying these VOC markers in breath samples through innovative statistical and machine learning techniques is an important task in lung cancer research. Therefore, we proposed an experimental approach for generation of VOC molecular concentration data using unique silicon microreactor technology and further identification and characterization of key relevant VOCs important for lung cancer detection through statistical and machine learning algorithms. We reported several informative VOCs and tested their effectiveness in multi-group classification of patients. Our analytical results indicated that seven key VOCs, including C_4_H_8_O_2_, C_13_H_22_O, C_11_H_22_O, C_2_H_4_O_2_, C_7_H_14_O, C_6_H_12_O, and C_5_H_8_O, are sufficient to detect the lung cancer patients with higher mean classification accuracy (92%) and lower standard error (0.03) compared to other combinations. In other words, the molecular concentrations of these VOCs in exhaled breath samples were able to discriminate the patients with lung cancer (n = 156) from the healthy smoker and nonsmoker controls (n = 193) and patients with benign pulmonary nodules (n = 65). The quantification of carbonyl VOC profiles from breath samples and identification of crucial VOCs through our experimental approach paves the way forward for non-invasive lung cancer detection. Further, our experimental and analytical approach of VOC quantitative analysis in breath samples may be extended to other diseases, including COVID-19 detection.

## Introduction

Lung cancer is the most common kind of cancer globally due to poor lifestyle and environmental pollution. In the USA, lung cancer has an incidence of 235,760 new cases with 131,880 deaths in 2020 and is by far the leading cause of cancer deaths among both men and women, making up almost 25% of all cancer fatalities [1]. Every year, a larger number of people die due to lung cancer than colon, breast, and prostate cancers combined [2]. The 5-year survival rate for lung cancer patients is relatively low compared to other diseases. Early detection of lung cancer is crucial to improve the survival of the patients [2]. For this purpose, many screening methods, including chest radiography, sputum cytology, low-dose spiral computer tomography, fluorescence bronchoscopy, and positron emission tomography have been used [3]. However, these procedures are complicated, expensive, and time-consuming, thus making them difficult for the poor or low-income patient groups. Therefore, lung cancer detection through breath analysis using volatile organic compounds (VOCs) can be a vital tool for its early detection in order to increase the survival chance.

VOCs are organic chemicals that exist in air, exhaled breath, *etc*. and have high vapor pressure at ambient temperature [4]. The analysis of VOCs in exhaled breath plays a vital role in the early detection of lung cancer [4]. Earlier studies showed that there are several thousand(s) of VOCs in human breath, *e*.*g*., formaldehyde (CH_2_O), acetaldehyde (C_2_H_4_O), acetone (C_3_H_6_O), 2-butanone (C_4_H_8_O), to name a few. So far, many VOCs have been identified and reported in breath samples of normal humans and patients with lung cancer [5]. For instance, in 1999, Phillips *et al*. reported over 3400 different VOCs present in exhaled normal human breath [6].

The combinations of some VOCs reported in the literature can comfortably discriminate the lung cancer patients from the healthy ones [7]. For instance, a recent study showed higher sensitivity of lung cancer detection among patients using the carbonyl VOCs present in the exhaled breath samples [8]. In other words, the molecular concentration data on the VOCs present in exhaled breath samples were collected from the patients to classify them into several groups, *i*.*e*., Healthy Control, Cancer, and Benign Nodule. In this direction, Bousamra *et al*. (2014) developed one quantitative analytical approach to differentiate early lung cancer from benign pulmonary nodules based on the molecular concentration of carbonyl compounds in exhaled breath samples [9]. In the existing analytical approaches, statistical methods including the t-test and Wilcoxon test were used to select the significant VOCs [7, 9]. These methods are univariate in nature and mostly ignore the inter-VOC relationships (*i*.*e*., relationship among the VOCs), and there is a chance of spurious association of VOC with the patient classes [16]. Further, Li *et al*. (2015) developed a technique utilizing quaternary amino-oxy coated silicon micro-reactors [4] for selective capture and quantification of the ketones and aldehydes in the air [10–12] and exhaled breath [7–9]. They used five classification models, including generalized partial least squares, support vector machines, random forests, linear and quadratic discriminant analyses to classify the patients into lung cancer and control groups based on exhaled breath data [4]. Sufficient studies in the literature indicated that the carbonyl compounds in exhaled breath play a significant role in the non-invasive detection of lung cancer [7–12]. Furthermore, the proper identification of key carbonyl compounds through statistical and machine learning techniques requires further advances.

In a typical breath sample study, the molecular concentration data on several hundred(s) of endogenous and exogenous VOCs are usually observed over patients. Sometimes, it may not be experimentally possible to monitor data on all the VOCs and further use them in lung cancer detection. In other words, among these hundred(s) of VOCs, all may not be required for the patient classification or the predictive model building process (*i*.*e*., training the machine learning models and later use them for class label predictions). Therefore, it is pertinent to select/identify a few metabolic VOCs related to lung cancer as key and significant features for cancer detection. The selection of important features (here metabolic VOCs) out of many VOCs is called feature selection in machine learning [13]. Further, it is essential to determine the number of significant VOCs (e.g., feature size or dimension of VOC data), which can be used in the training of the classification model to predict the class type of lung cancer patients. The selection of significant VOCs will save the precious time and cost of data generation for all VOCs present in the breath samples. In other words, the researchers can focus on few VOCs instead of generating data on all the VOCs present in breath samples of the patients.

Therefore, in this study, we endeavor to classify the lung cancer patients based on the VOC molecular concentration data. We present an experimental approach for lung cancer prediction using the carbonyl VOCs present in breath samples. The VOC molecular concentration data are generated from the breath samples of the 414 subjects (156: lung cancer; 65: benign and 193: healthy control) through the unique silicon microreactor technology [8]. The breath samples are collected following the experimental protocol approved by the Institutional Review Board (IRB) at the University of Louisville, USA. We also present an analytical approach involving relevant VOC selection and further used them in lung cancer patient classification model training. This approach of VOC selection is statistically sound, robust, and does not require any probability distributional assumptions about the VOC data (for VOCs testing and selection). Further, we identified several informative VOCs present in exhaled breath samples to detect lung cancer patients. For instance, the developed models provided sufficient classification accuracy for lung cancer detection with a minimum of three VOCs. Also, we studied the effect of the various VOC combinations on the classification of lung cancer patients. Moreover, our developed experimental approach can be applied to detect COVID-19 patients using VOC data from the exhaled breath samples.

The remainder of the paper is organized as follows: (i) the material and methods section deals with detail protocols for the data generation, description of methodology; (ii) the results and discussion section mainly deals with presentation of obtained results along with their discussion; and (iii) the conclusion section summarizes the manuscript along with its future scope.

## Materials and methods

### Breath sampling and data generation

This study recruited 156 patients with untreated lung cancer, 65 patients with benign pulmonary nodules, and 193 healthy control subjects to provide exhaled breath samples. The detailed subject demographic characteristics, disease information, and breath analysis data have been published [8]. In brief, there were 103 patients with early stages (Stages 0, I, and II) of lung cancer and 53 patients with late stages of lung cancer. Most lung cancer patients were current or former smokers (149). The healthy controls included 113 current or former smokers and 80 never smokers. The average ages of the lung cancer patients, patients with benign pulmonary nodules, and healthy subjects were 65.1, 54.2, and 49.4 years, respectively. The male percentages of these three subgroups were 51.9%, 49.2%, and 55.7%, respectively.

The detailed research protocol for the collection of exhaled breath samples was approved by the IRB, University of Louisville, USA (IRB #15.0711). The healthy control subjects were recruited from patient family members who were free of lung cancer or other chronic pulmonary disease. All patients with pulmonary nodules were recruited in the James Graham Brown Cancer Center and Jewish Hospital at the University of Louisville, USA. The diagnostic predictions from these breath analyses were confirmed by clinical diagnoses using following-up the CT scans, positron emission tomography scans or pathology of biopsy or surgically resected specimens.

**Fig 1:**
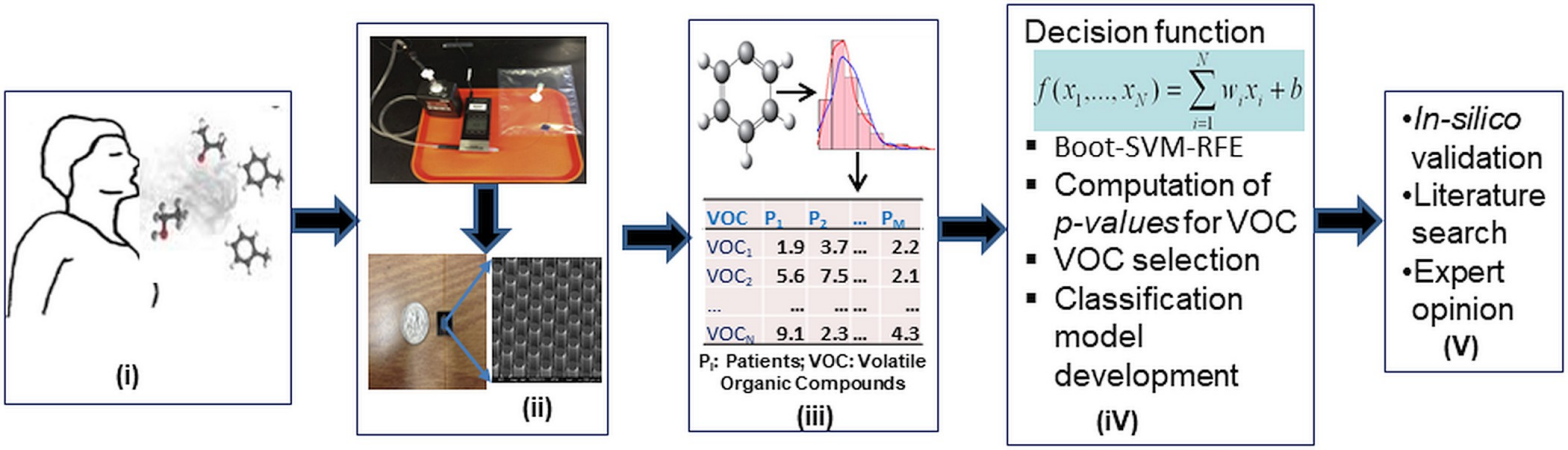
Outline of the experimental approach used in this study. Various steps undertaken in this study are shown in flow chart form. The various steps include (i) capturing of exhaled breath; (ii) capturing VOCs present in exhaled breath samples; (iii) generation of molecular concentration data (nmole/L) of VOCs through bioassay mass-spectrometry technique; (iv) feature (VOC) selection and classification model training and its application for lung cancer detection; and (v) validation of the selected VOCs through classification accuracy, literature search, and expert opinion.

Exhaled breath samples were collected in one-liter Tedlar bags (Sigma-Aldrich, USA) through normal exhalation allowing for the collection of a mixture of alveolar and tidal breath in one exhaled breath. Ambient clinic exam room air samples (1 L) were also collected to serve as a control of background carbonyl compounds in the collection room. Our previous studies have examined the detailed method of breath sample collection, evacuation of breath samples through the micro-reactors, and analysis of the samples [4, 7–9]. In a brief description, the subjects directly exhaled the breath into Tedlar bags through the Teflon tube from the mouth to provide one liter exhaled breath samples, thus providing a non-invasive collection technique that the patients readily accepted. After the collection of exhaled breath, the Tedlar bags were directly connected to the silicon micro-reactors through silica capillary tubes and septa. A vacuum was applied to draw the collected VOCs from the Tedlar bags through the fabricated microreactor [10–12] at a flow rate of 5 mL/min. The microreactor has thousands of micropillars coated with 2-(aminooxy)-N, N, N-trimethyl-ethanammonium (ATM) iodide. After complete deflation of the sample bags, ATM and ATM adducts were eluted by flowing methanol (~100 μL) from a pressurized vial through the microreactor and into a collection vial [10]. The eluent methanol solutions were directly analyzed using a hybrid linear ion trap-Fourier transform-ion cyclotron mass spectrometry (FT-ICR-MS) instrument (Finnigan LTQ-FT, Thermo Electron, Bremen, Germany) equipped with a TriVersa NanoMate ion source (Advion BioSciences, Ithaca, NY) and a nano-electrospray chip (inner nozzle diameter 5.5 μm). A 5 μL methanol solution of a known amount of 5 nmol of ATM–acetone-d6 adduct was added to each eluted methanol sample as an internal reference of FT-ICR-MS. The amount of captured carbonyl compounds was then determined by comparing the FT-ICR-MS signal abundance of ATM−acetone-d6 with those of other ATM−carbonyl adducts. The concentration of each compound in exhaled breath detected by FT-ICR-MS was then calculated from the amount of the captured carbonyl compounds with in terms of nanomoles per liter (nmole/L). The microreactor’s carbonyl capture efficiencies and validity of the analyses have been characterized in our previous studies [10–12]. A flow chart of the bioassay for generating data is displayed in Fig 1. Further, the workflow of the proposed experimental approach, including data generation, feature selection, and the classification model development is also illustrated in Fig 1.

This study was approved by the IRB at the University of Louisville (IRB #15.0711). An informed written consent form was reviewed by the subjects for participation. A signed consent form was obtained from the subjects before they participated in the study. There was no minor recruited for the study.

### Notation

Let, X_*N* × *M*_ = [*x*_*i*, *m*_] be the VOC data matrix, where *x*_*i*, *m*_ represents the observed measurement of the *i*^*th*^ (*i* = 1, 2, …, *N*) VOC for the *m*^*th*^ (*m* = 1, 2, …, *M*) patient; ***x***_*m*_ be the *N-*dimensional vector of observed values of *N* VOCs for *m*^*th*^ patient; *y*_*m*_ be the outcome variable for target class label of *m*^*th*^ patient (*i*.*e*., control and lung cancer) and takes values {+1, -1} for lung cancer and control conditions respectively; *M*_1_ and *M*_2_ be the number of patients in lung cancer and control classes respectively (*M*_1_+*M*_2_ = *M*).

### Support Vector Machine-Recursive Feature Elimination

Support Vector Machine-Recursive Feature Elimination (SVM-RFE) can be used for the selection of relevant VOCs (in a two group case) from the lung cancer VOC data [14, 15]. Let, {***x***_*m*_, *y*_*m*_} *ϵ R*^*N*^ × {−1, 1} be the input given to the Support Vector Machine (SVM) model. Here, we wish to find a hyperplane that divides the patients for lung cancer (*y*_*m*_ = 1) from that of control class (*y*_*m*_ = −1) in such a way that the distance between the hyperplane and the closest point is maximum (Supplementary Document S1 in S1 File). Then the hyperplane can be written as:

k.x+b=0 (1)

where, *k*_*i*_ and *b* are the weight of *i*^*th*^ VOC and bias, respectively. Here, we assume that the patients for the two classes are linearly separable. In other words, we can select two parallel support hyperplanes that separate the lung cancer and control classes in such a way that the distance between these two planes is maximized (Supplementary Document S1 in S1 File).

For the lung cancer class (*y*_*m*_ = 1), the supporting hyperplane can be written as:

k.x+b=1 (2)

Here, the Eq 2 only holds good for the ***x*** which are support vectors (i.e., points closest to the separating hyperplane, Eq 1).

For control class (*y*_*m*_ = −1), the support hyperplane can be written as:

k.x+b=−1 (3)

Now, we assume that every point must lie on either side of the respective support hyperplanes (Eq 2 and Eq 3) (their distance to the separating hyperplane is at least same as the distance between the support vectors and the separating hyperplane), which can be expressed as:

$$y_{m}(k.x_{m}+b)\geq 1\;\forall \;m=1,2,\ldots ,M$$ (4)

We wish to maximize the distance between the lung cancer and control support hyperplanes given in Eqs 2 and 3, respectively. Here, special care must be taken to prevent any data points from falling in between these two support hyperplanes.

To maximize the distance between the support hyperplanes (Supplementary Document S1 in S1 File), we need to minimize $\frac{{\left\|k\right\|}^{2}}{2}$ under the constraint of Eq 4. Mathematically, the objective function can be written as:

$$L_{p}=\underset{k}{\min}\left[\frac{{\left\|k\right\|}^{2}}{2}+\sum_{m=1}^{M}\varphi_{m}\{1-y_{m}(k.x_{m}+b)\}\right]\;\forall \;m=1,2,\ldots ,M$$ (5)

where, *φ*_*m*_ (≥ 0) are Lagrange multipliers. The constraint on *φ*_*m*_ follows from the fact that the constraint is an inequality. Here, *k*_*i*_’s are obtained by minimizing the objective function in Eq 5. The objective function (Eq 5) was optimized with respect to *k*_*i*_, *b* and the following expressions are obtained.


$$\frac{\partial L_{p}}{\partial k}=\|k\|-(\sum_{m=1}^{M}\varphi_{m}y_{m}x_{m})=0\;and\;\frac{\partial L_{p}}{\partial b}=\sum_{m=1}^{M}\varphi_{m}y_{m}=0$$ (6)

The value of ***k*** can be obtained through solving the system of linear equations given in Eq 6 and is expressed as:

$$k=\sum_{m=1}^{M}\varphi_{m}y_{m}x_{m}\;with\;\sum_{m=1}^{M}\varphi_{m}y_{m}=0\;and\;\varphi_{m}\geq 0$$ (7)

Here, one also need to solve for *φ*_*m*_, but this cannot be done by solving for Eq 6 when its gradient is zero, thus one need to take into account the constraint that *φ*_*m*_≥0. Therefore, SVMs cannot be solved via a linear system of equations. Rather, optimization algorithms (e.g., gradient descent) are commonly used in SVM-based tools. For this purpose, we executed the *svm* function implemented in e1071 R package.

The *k*_*i*_^2^ (≥ 0) (*i*.*e*., square of the *i*^*th*^ element of ***k*** in Eq 7) is used as a metric for the ranking of the VOCs in the data [15] to evaluate the impact of the *i*^*th*^ VOC on patients’ classification [16]. In the SVM-RFE technique, VOCs are eliminated with the smallest *k*_*i*_^2^ iteratively in a backward elimination manner and ranked VOC list is prepared at the end [15, 17]. Here, the backward elimination means training the SVM-based machine learning models iteratively after removing the least significant VOC at each step. Moreover, most feature selection methods, such as SVM-RFE, are sensitive to slight permutations of the class labels [18]. The ranking of VOCs may also lead to the selection of spuriously associated VOCs and make the selection process unreliable [13, 19, 20]. Therefore, it is essential to select VOCs based on statistical testing instead of their ranks. For this purpose, we used the Bootstrap SVM-RFE (Boot-SVM-RFE) technique developed by Das *et al*. (2017) to select the most relevant VOCs for patient classification [19]. The Boot-SVM-RFE method is briefly described in the following section.

### Bootstrap SVM-RFE

In the usual supervised setting, the *M* patients/samples (as columns) in the ***X***, data matrix, either belonging to lung cancer or control, can be considered subjects/units in a population model, as shown in Eq 8.

$$\left(x_{1},y_{1}),(x_{2},y_{2}),\ldots ,(x_{m},y_{m}),\ldots ,(x_{M-1},y_{M-1}),(x_{M},y_{M}\right)$$ (8)

where, ***x***_*m*_ is the *N-*dimensional vector of the VOC measurements of the *m*^*th*^ patient/sample and *y*_*m*_ the class label (e.g., cancer *vs*. control) *m*^*th*^ patient/sample. Here, it is assumed that the *M* measurements of the different patients/samples (i.e., ***x***_*m*_ ∀ *m* = 1,2,…,*M*) are independent and identically distributed (iid), but the VOCs within the same sample may be correlated. In the bootstrap procedure, *M* units are randomly drawn from *M* population units in Eq 8 with replacement to constitute a bootstrap data matrix, *i*.*e*., $X_{N\times M}^{\left(b\right)}$ (*M* units serve as *M* columns of ***X***). This process is repeated *B* times to get *B* bootstrap data matrices, *i.e.,* $X_{N\times M}^{\left(1\right)},X_{N\times M}^{\left(2\right)},\ldots ,X_{N\times M}^{\left(b\right)},\ldots ,X_{N\times M}^{\left(B\right)}$. Here, *B* (*i*.*e*., number of bootstrap samples) depends on several factors, such as the number of units in the population model in Eq 8 [15, 19]. So, we set *B* = 500 as the literature showed that the number of bootstrap samples required for obtaining the distribution of the test statistic(s) must be sufficiently large [18, 21]. Furthermore, the values of *M* for different classification problems including Cancer *vs*. Control, Cancer *vs*. Benign, Benign *vs*. Control, (Benign + Cancer) *vs*. Control, and Cancer *vs*. (Benign + Control) are 349, 221, 258, 414, and 414 respectively.

The *B* bootstrap data matrices are given as input to the SVM-RFE technique to compute the *k*_*i*_^2^ scores (given in Eq 7), and subsequently, VOC ranking was performed on each of the *B* bootstrap data matrices. Let, *P*_*ib*_ be a random variable (*rv*) that indicates the position of *i*^*th*^ VOC (i.e., ranks obtained from the SVM-RFE) in the *b*^*th*^ bootstrap data matrix. Then, another *rv* can be defined based on *P*_*ib*_ (without loss of generality), given as:

$$R_{ib}=\frac{N+1-P_{ib}}{N};\;0\leq R_{ib}\leq 1$$ (9)

where, *R*_*ib*_ in Eq 9 is the rank score of *i*^*th*^ (*i* = 1, 2, …, *N*) VOC in the *b*^*th*^ (*b* = 1, 2, …, *B*) bootstrap data matrix. Here, it may be noted that the distribution of the rank scores of VOCs, computed from a bootstrap data matrix, is symmetric around the median value, 0.5 (as rank scores are functions of ranks).

To decide whether *i*^*th*^ VOC is relevant or not for the patient classification, the following null hypotheses are framed.

*H*_0_: *R*_*i*_ ≤ 0.5 (*i*^*th*^ VOC is not so relevant to patient classification)

*H*_1_: *R*_*i*_ > 0.5 (*i*^*th*^ VOC is relevant to patient classification)

where, *R*_*i*_ is the rank score for *i*^*th*^ VOC over all possible bootstrap samples.

To obtain the distribution of test statistic under *H*_*0*_, we defined another *rv Z*_*ib*_, as:

$$Z_{ib}=\left\{ \begin{array}{l} 1\;if\;{(R}_{ib}-0.5)>0\\ 0\;if\;{(R}_{ib}-0.5)\leq 0 \end{array}\right.$$ (10)

Let *r*_*ib*_ be another *rv* representing the rank assigned to (*R*_*ib*_ −0.5) (after arranging in ascending order of their magnitudes). In other words, for each row (which denotes the VOC), (*R*_*ib*_ −0.5) was computed and corresponding rank, *r*_*ib*_, was assigned to (*R*_*ib*_ −0.5.

To test *H*_*0*_ *vs*. *H*_*1*,_ the test statistic for *i*^*th*^ VOC, *W*_*i*_, is developed and is given as:

$$W_{i}=\sum_{b=1}^{B}U_{ib}$$ (11)

where, *U*_*ib*_ = *Z*_*ib*_*r*_*ib*_.

In other words, *W*_*i*_ (Eq 11) is the sum of the ranks of positive (*R*_*ib*_ −0.5) for *i*^*th*^ VOC over *B* bootstrap samples. Further, *U*_*ib*_ in Eq 11 is a Bernoulli *rv*, and its probability mass function can be given as:

$$P[U_{ib}=u_{ib}]=\left\{ \begin{array}{l} \frac{1}{2}\;if\;u_{ib}=0\\ \frac{1}{2}\;if\;u_{ib}=b \end{array}\right.$$ (12)

Here, the expected value and variance of *W*_*i*_ in Eq 11 under *H*_*0*_ can be obtained as:

$$E(W_{i})=\sum_{b=1}^{B}{E(U}_{ib})=\frac{1}{2}\sum_{b=1}^{B}b=\frac{B(B+1)}{4}$$ (13)

The variance of *W*_*i*_ becomes: $V(W_{i})=\sum_{b=1}^{B}\left\{E(U_{ib}^{2})-{E(U_{ib})}^{2}\right\}$

$$=\frac{1}{4}\sum_{b=1}^{B}b^{2}=\frac{B(B+1)(2B+1)}{24}$$ (14)

As *B* is sufficiently large, then under the central limit theorem, the distribution of *W*_*i*_, given in Eq 11, becomes:

$$Z_{i}=\frac{W_{i}-E(W_{i})}{\sqrt{V(W_{i)}}}\overset{d}{\to }N(0,1)$$ (15)

Through Eq 15, the *p-value* for *i*^*th*^ (*i =* 1, 2, …, *N*) VOC was computed, and similarly, this testing procedure was repeated for the remaining *N*-1 VOCs. In other words, the above statistical test was repeated for *N* times to compute the statistical significance values for the VOCs.

Let, *p*_1_, *p*_2_,…,*p*_*N*_ be the corresponding *p-values* for all the VOCs, and *α* be the desired significance level. Hence, we employed Hochberg’s procedure [1] to correct the multiple testing problem and computed the adjusted (*adj*.) *p-values* for the VOCs. The algorithm for Hochberg’s procedure [22] is as follows:

First, the *p-values* of the VOCs were sorted in increasing order of their magnitude, shown as: *p*_(1)_, *p*_(2)_,…,*p*_(*N*)_, where, *p*_(.)_ is the rank ordered *p-value* and (*i*) stands for the *i*^*th*^ ordered value (*i*.*e*., *p*_(1)_: smallest *p-value*, *p*_(2)_: second smallest *p-value* and so on).

**Step 1** . If *p*_(*N*)_>α, then retain corresponding null hypothesis and go to the next step. Else reject it and stop.
**Step *i*** = 2,3,…, *N*−1. If *p*_(*N*−*i*+1)_>α/*i*, then retain the corresponding null hypothesis and go to the next step. Else reject all remaining hypotheses and stop.
**Step *N***. If *p*_(1)_>α/*N*, then retain the corresponding null hypothesis. Else reject it.

Now, the *adj*. *p-values* are given recursively beginning with the largest *p-value* [1]:

$${\tilde{p_{}}}_{\left(i\right)}=\left\{ \begin{array}{l} \;p_{\left(i\right)}\;if\;i=N\\ \min({\tilde{p_{}}}_{\left(i+1\right)},(N-i+1)p_{\left(i+1\right)})\;if\;i=N-1,\ldots ,1 \end{array}\right.$$ (16)

**Fig 2:**
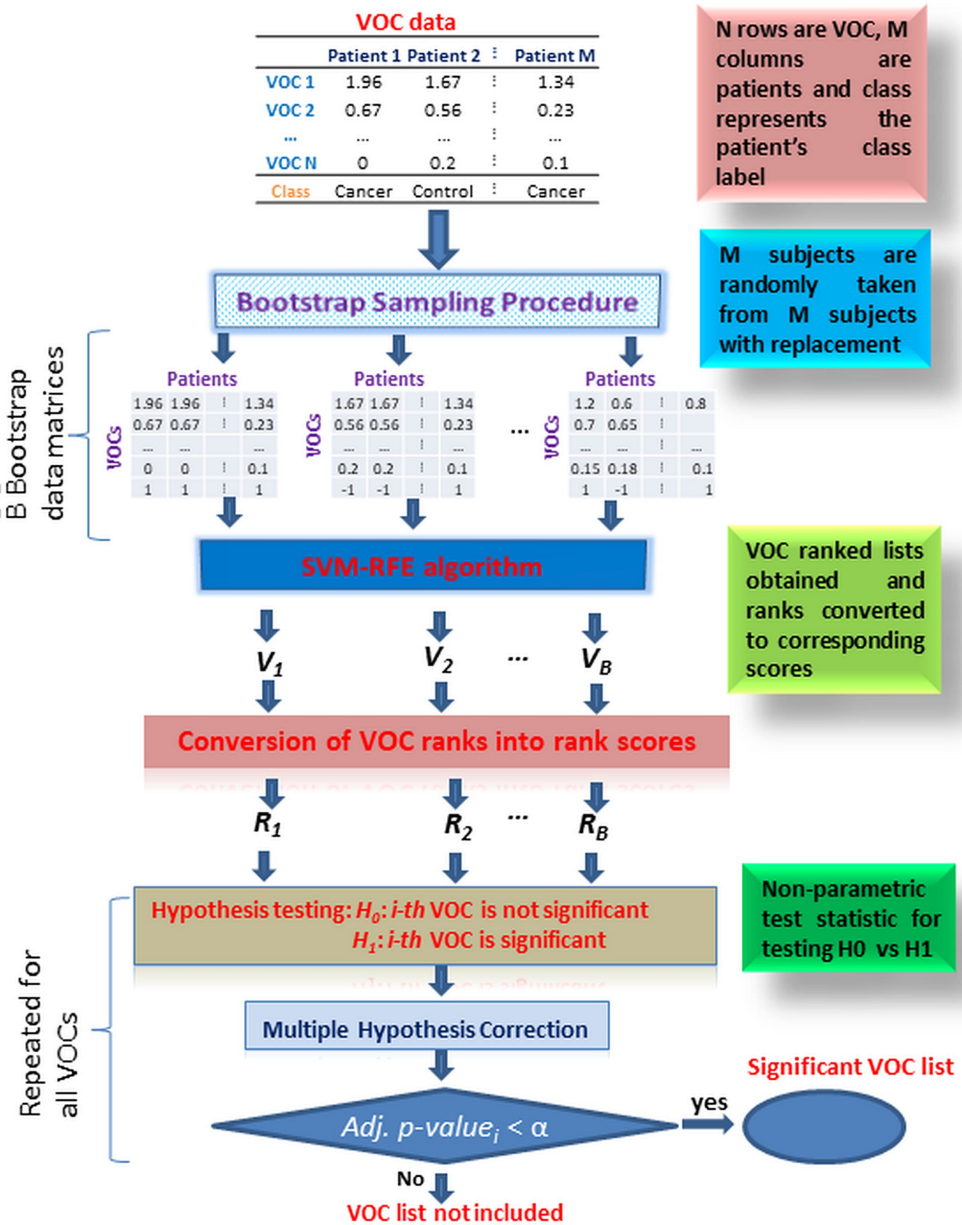
Outline of the Boot-SVM-RFE technique for significant VOC selection. Data matrix has *N* rows as VOCs with concentrations of nmole/L and M columns as patients/samples. B is the number of bootstrap samples drawn from the data matrix. *V*_*b*_ and *R*_*b*_’s are the VOC ranked list and VOC rank-score for the *b*^*th*^ bootstrap sample. Null hypothesis represents *i*^*th*^ VOC is not important for patients’ classification. α is the desired level of significance.

Based on the computed *adj*. *p-values*, the relevant VOCs were selected from the data. In other words, a lesser value of *adj*. *p-value* indicates more relevance of the VOC for the patient classification and *vice-versa*. Similarly, this procedure was applied to select the significant VOC for the patient classification into classes, such as benign *vs*. control, (lung cancer + benign) *vs*. control, (control + benign) *vs*. lung cancer. The outline and key analytical steps of the VOC selection process are shown in Fig 2. The effects of the significant VOCs on the patient classification were studied through an SVM classifier (with linear basis function). Under this setting, the VOCs (and their molecular concentration data) were given as inputs to the SVM classifier to compute the classification-based performance metrics. Further, impacts of the VOCs on the classification of patients under five different cases, including Case I: Cancer *vs*. Control; Case II: Cancer *vs*. Benign; Case III: Benign *vs*. Control; Case IV: (Benign + Cancer) *vs*. Control; and Case V: (Control + Benign) *vs*. Cancer were assessed through performance metrics, such as mean classification accuracy (CA) and standard error (SE) in CA computed through a varying sliding window size technique [18, 19]. Here, we used this technique to study the importance of rankings of VOCs (obtained from a feature selection method, *e*.*g*., Boot-SVM-RFE) through training a classification model. In other words, the sliding windows are VOC intervals that literally "slide" across the whole VOC list, preferably by some constant distance and CA is computed for each sliding window. Sliding windows can overlap or mutually exclusive. A brief description about the varying sliding window size technique is given in Supplementary Document S2 in S1 File. Then, the mean CA and SE in CA were computed over the sliding windows. In other words, the VOC set (of size *n* (*n* ≤ *N*)), which provides maximum discrimination between the subjects/patients of 2 groups through CA, will be considered as the optimal size of the VOC set. The expressions for mean CA and SE in CA computed through varying sliding window size technique are given in Eqs 17 and 18.


μCA=(∑k=1KCAk)K (17)

SECA=∑k=1K(CAk−μCA)2K(K−1) (18)

The total number of windows denoted as *K* in Eqs 17 and 18 can be defined in Eq 19.

K=(n−S)/L (19)

where, *n* is the size of the VOC set, *S* is the size of the windows (*i*.*e*., size refers to the number of ranked VOCs), and *L* is the sliding length. The values of *n*, *S*, L, and *K* are given in Supplementary Document S2 in S1 File. The optimal size of the VOC set was computed through training the SVM-based classification model *(i*.*e*., calculating the indices given in Eqs 17 and 18) using the five-fold cross-validation technique. The outline and workflow of the Boot-SVM-RFE for the key VOC selection are shown in Fig 2.

## Results and discussion

We observed the carbonyl VOCs having carbon atoms ranging from one (formaldehyde: CH_2_O) to thirteen (tridecanal: C_13_H_26_O) in exhaled breath samples of healthy controls, benign nodules, and lung cancer patients. Through FT-ICR-MS technology, the molecular concentration data on VOCs with chemical formulas CH_2_O, C_2_H_4_O, C_3_H_6_O, C_4_H_8_O, C_5_H_10_O, C_6_H_12_O, C_7_H_14_O, C_8_H_16_O, C_9_H_18_O, C_10_H_20_O, C_11_H_22_O, C_12_H_24_O, C_13_H_26_O, C_4_H_8_O_2_, C_2_H_4_O_2_, C_3_H_4_O, C_6_H_10_O_2_, C_9_H_16_O_2__HNE, C_3_H_4_O_2_, C_4_H_6_O_2_, C_4_H_6_O, C_4_H_4_O_2_, C_5_H_8_O, C_7_H_6_O, C_7_H_11_O, C_13_H_22_O, and C_15_H_10_O, were observed for healthy controls, benign nodules, and lung cancer patients. The common names of these VOCs are provided in Supplementary Document S3 in S1 File. The FT-ICR-MS spectra in Fig 1 showed the capturing of typical relative abundances of these VOCs in breath samples of lung cancer patients, benign, and healthy controls. Chemical structure-wise, isomeric ketones and aldehydes are indistinguishable by direct infusion of one-dimensional FTICR-MS. However, the measured molecular weight at a resolving power of 200,000 provides their accurate chemical formulas. Separation and structure identification of some important isometric ketones and aldehydes was done through FT-ICR-MS/MS and GC–MS technologies. Further, the summary statistic(s) of the 27 considered VOCs for three different patient classes, *i*.*e*., control, benign, and lung cancer, are given in S1 Table. The mean values of the VOCs, including C_3_H_6_O, C_2_H_4_O, *etc*., are higher than others across all the patient classes, which indicated their higher average concentrations in breath samples (S1 Table).

**Fig 3:**
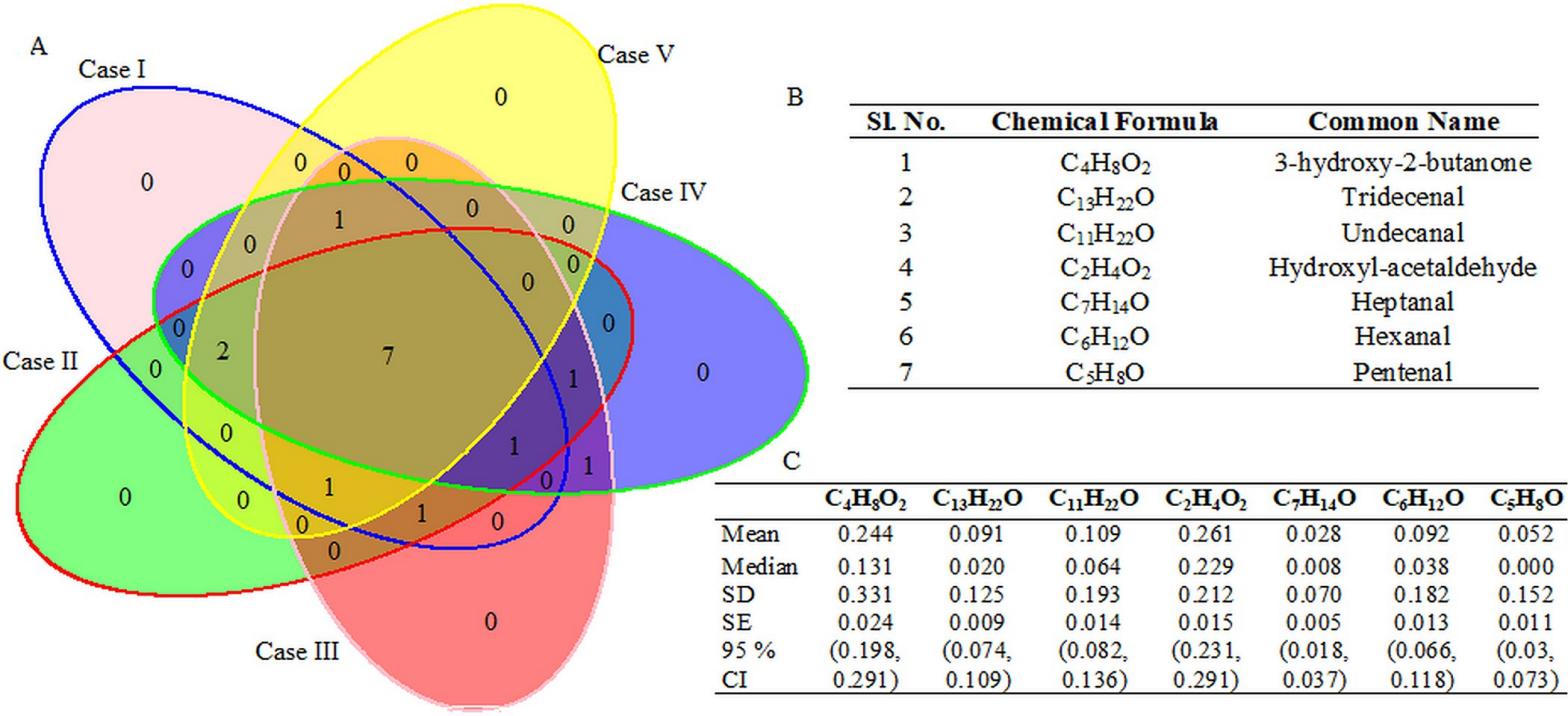
Identification and characterization of key common VOCs for the patients’ classification under all the five cases. (A) Venn diagram for the significant VOCs identified through the Boot-SVM-RFE technique. Significant VOCs are identified by setting the threshold for *p-values* at 10^−15^. Through this, 15, 15, 15, 13 and 13 significant VOCs are selected for the Case I, Case II, Case III, Case IV, and Case V, respectively. Case I: Lung cancer vs. Control; Case II: Cancer vs. Benign; Case III: Benign vs. Control; Case IV: (Benign + Cancer) vs. Control; Case V: (Control + Benign) vs. Cancer. (B) Characterization of the common significant VOCs. (C) Summary statistics of the seven significant VOCs with mean and median concentrations (nmole/L) over the whole sample (n = 414). SD: Standard deviation; SE: Standard error; CI: Confidence Interval.

All the 27 VOCs may not be considered as biomarkers for lung cancer detection; therefore, we used the Boot-SVM-RFE technique to identify the significant VOCs for various combinations of patient classes. Here, five different classes were used, *i*.*e*., Case I: Cancer (156) *vs*. Control (193); Case II: Cancer (156) *vs*. Benign (65); Case III: Benign (65) *vs*. Control (193); Case IV: (Benign + Cancer) (221) *vs*. Control (193); Case V: (Control + Benign) (258) *vs*. Cancer (156) (Fig 3). Through the Boot-SVM-RFE, the statistical significance values (*p-values*) for the VOCs were computed for five different classification problems and shown in Table 1 and S2 Table. For instance, the VOCs, including C_4_H_8_O, C_6_H_12_O, C_7_H_14_O, C_8_H_16_O, *etc*., were found to be statistically significant (at 1% level of significance) for all the cases of classification (Table 1). This finding indicated that these VOCs can be used as biomarkers for the patient classification. Broadly, we found 16 VOCs as statistically significant, at least in one case of patients’ classification (Table 1). The summary statistic(s) for these key VOCs for the three patients’ classes are shown in Table 2. The co-efficient variation of the VOCs, including C_3_H_4_O, C_6_H_10_O_2_, C_9_H_16_O_2_, C_5_H_8_O, C_7_H_11_O, and C_13_H_22_O, are quite high as compared to others (Table 2). This observation indicated that the molecular concentrations of these VOCs have greater dispersion levels than others across all the classes (Table 2). Similar interpretations can be made for other VOCs.

**Table 1:** Effects of the VOCs on lung cancer classifications.

	Case I		Case II		Case III		Case IV		Case V	
VOCs	Adj. Pval	FDR	Adj. Pval	FDR	Adj. Pval	FDR	Adj. Pval	FDR	Adj. Pval	FDR
CH_2_O	NS	NS	NS	NS	NS	NS	NS	NS	NS	NS
C_2_H_4_O	NS	NS	NS	NS	NS	NS	NS	NS	NS	NS
C_3_H_6_O	NS	NS	NS	NS	NS	NS	NS	NS	NS	NS
C_4_H_8_O	1.58E-82	5.6E-83	8.20E-31	1.2E-31	1.06E-06	1.5E-07	1.05E-72	2.4E-73	3.56E-80	1.28E-80
C_5_H_10_O	NS	NS	NS	NS	NS	NS	NS	NS	NS	NS
C_6_H_12_O	2.43E-49	3.8E-50	3.54E-45	5.9E-46	2.23E-15	3.1E-16	7.38E-25	1.1E-25	8.44E-45	1.42E-45
C_7_H_14_O	2.26E-74	4.2E-75	4.46E-55	9.1E-56	5.7E-56	1.1E-56	7.23E-70	1.5E-70	8.24E-50	1.51E-50
C_8_H_16_O	1.83E-23	2.6E-24	2.96E-15	4.2E-16	4.60E-18	6.5E-19	9.74E-14	1.3E-14	7.55E-05	1.05E-05
C_9_H_18_O	NS	NS	0.4940	0.0694	NS	NS	NS	NS	NS	NS
C_10_H_20_O	NS	NS	NS	NS	1.31E-30	2.1E-31	4.82E-21	6.8E-22	NS	NS
C_11_H_22_O	2.41E-80	6.8E-81	9.94E-15	1.4E-15	1.94E-81	6.9E-82	2.39E-82	8.6E-83	2.49E-54	5.10E-55
C_12_H_24_O	NS	0.3069	1.78E-71	6.4E-72	1.44E-59	3.4E-60	7.01E-29	1.1E-29	0.648013	0.089725
C_13_H_26_O	3.72E-27	5.6E-28	3.76E-52	6.9E-53	9.68E-20	1.4E-20	4.94E-12	6.8E-13	9.41E-28	1.41E-28
C_4_H_8_O_2_	1.58E-82	5.7E-83	4.21E-67	1.2E-67	1.67E-82	8.6E-83	1.64E-82	8.5E-83	1.84E-82	1.21E-82
C_2_H_4_O_2_	1.56E-77	3.7E-78	4.23E-31	6.3E-32	3.91E-69	1.1E-69	3.40E-64	5.7E-65	8.66E-65	2.03E-65
C_3_H_4_O	4.43E-25	6.4E-26	1.80E-32	2.8E-33	5.44E-24	8.1E-25	1.33E-15	1.8E-16	3.20E-08	4.51E-09
C_6_H_10_O_2_	7.01E-76	1.4E-76	8.05E-77	4.2E-77	0.037348	0.00571	5.06E-77	1.4E-77	2.34E-82	1.21E-82
C_9_H_16_O_2_	1.25E-54	2.1E-55	6.15E-12	8.5E-13	5.90E-57	1.2E-57	3.31E-66	6.1E-67	6.07E-21	8.76E-22
C_3_H_4_O_2_	NS	NS	NS	NS	NS	NS	NS	NS	NS	NS
C_4_H_6_O_2_	NS	NS	NS	NS	NS	NS	NS	NS	NS	NS
C_4_H_6_O	NS	NS	NS	NS	NS	NS	NS	NS	NS	NS
C_4_H_4_O_2_	NS	NS	NS	NS	NS	NS	NS	NS	NS	NS
C_5_H_8_O	1.75E-14	2.4E-15	5.06E-82	5.1E-82	2.12E-53	3.6E-54	7.32E-29	1.1E-29	3.83E-33	6.04E-34
C_7_H_6_O	NS	NS	NS	NS	NS	NS	NS	NS	NS	NS
C_7_H_11_O	0.887738	0.12229	3.57E-09	4.9E-10	NS	NS	NS	NS	0.207624	0.028601
C_13_H_22_O	1.58E-82	5.7E-83	3.50E-64	8.21E-65	1.67E-82	8.7E-83	1.64E-82	8.54E-83	2.24E-76	6.31E-77
C_15_H_10_O	NS	NS	NS	NS	NS	NS	NS	NS	NS	NS

Case I: Cancer (156) vs. Control (193); Case II: Cancer (156) vs. Benign (65); Case III: Benign (65) vs. Control (193); Case IV: (Benign + Cancer) (221) vs. Control (193); Case V: (Control + Benign) vs. Cancer. Adj. P-val.: Adjusted p-values for multiple hypothesis correction; FDR: False Discovery Rate; VOC: Volatile Organic Compounds. NS: highly non-significant

**Table 2:** Summary statistic(s) of the important VOCs for different patient populations.

	C_4_H_8_O	C_6_H_12_O	C_7_H_14_O	C_8_H_16_O	C_10_H_20_O	C_11_H_22_O	C_12_H_24_O	C_13_H_26_O	C_4_H_8_O_2_	C_2_H_4_O_2_	C_3_H_4_O	C_6_H_10_O2	C_9_H_16_O_2_	C5H8O	C_7_H_11_O	C_13_H_22_O
					Control (n = 193)											
Mean	1.400	0.058	0.014	0.052	0.671	0.112	0.112	0.046	0.087	0.186	0.034	0.003	0.014	0.052	0.119	0.013
Median	1.370	0.021	0.007	0.025	0.516	0.089	0.078	0.012	0.073	0.181	0.000	0.001	0.000	0.000	0.000	0.000
SD	0.611	0.071	0.019	0.085	0.614	0.123	0.139	0.082	0.079	0.115	0.201	0.008	0.060	0.123	0.265	0.046
Min	0.200	0.000	0.000	0.000	0.007	0.000	0.000	0.000	0.000	0.001	0.000	0.000	0.000	0.000	0.000	0.000
Max	2.842	0.394	0.143	0.655	5.248	0.979	1.271	0.539	0.548	0.656	2.553	0.091	0.557	0.946	2.170	0.435
CV	0.44	1.22	1.41	1.61	0.91	1.10	1.25	1.77	0.91	0.62	5.88	2.55	4.36	2.36	2.23	3.44
SE	0.044	0.005	0.001	0.006	0.044	0.009	0.010	0.006	0.006	0.008	0.014	0.001	0.004	0.009	0.019	0.003
95% CI	(1.314, 1.486)	(0.048, 0.068)	(0.011, 0.016)	(0.041, 0.064)	(0.585, 0.758)	(0.094, 0.129)	(0.092, 0.131)	(0.035, 0.058)	(0.075, 0.098)	0.17, 0.202	(0.006, 0.063)	(0.002, 0.004)	(0.005, 0.022)	(0.035, 0.069)	(0.081, 0.156)	(0.007, 0.020)
					Benign Pulmonary Nodule (n = 65)											
Mean	1.941	0.060	0.037	0.062	0.973	0.095	0.114	0.059	0.222	0.227	0.012	0.005	0.009	0.0675	0.1358	0.1568
Median	1.720	0.024	0.004	0.061	0.943	0.058	0.088	0.021	0.142	0.202	0.000	0.001	0.001	0.000	0.007	0.137
SD	0.930	0.117	0.134	0.047	0.682	0.141	0.151	0.125	0.277	0.163	0.044	0.008	0.022	0.151	0.333	0.131
Min	0.290	0.000	0.000	0.000	0.024	0.001	0.001	0.000	0.000	0.000	0.000	0.000	0.000	0	0	0
Max	4.570	0.841	1.016	0.216	3.308	0.854	0.929	0.739	1.830	0.765	0.315	0.053	0.102	0.723	1.832	0.393
CV	0.48	1.95	3.65	0.76	0.70	1.48	1.32	2.11	1.25	0.72	3.62	1.81	2.53	2.23	2.46	0.83
SE	0.115	0.014	0.017	0.006	0.085	0.017	0.019	0.015	0.034	0.020	0.005	0.001	0.003	0.019	0.041	0.016
95% CI	(1.715, 2.168)	(0.032, 0.088)	(0.004, 0.069)	(0.051, 0.074)	(0.807, 1.138)	(0.061, 0.129)	(0.078, 0.151)	(0.029, 0.089)	(0.155, 0.289)	(0.187, 0.267)	(0.001, 0.023)	(0.003, 0.007)	(0.003, 0.014)	(0.031, 0.104)	(0.055, 0.217)	(0.125, 0.189)
				Lung Cancer (n = 156)												
Mean	3.273	0.147	0.041	0.076	1.033	0.111	0.146	0.064	0.449	0.368	0.014	0.016	0.007	0.045	0.143	0.161
Median	3.020	0.065	0.018	0.065	0.816	0.058	0.101	0.020	0.308	0.325	0.000	0.007	0.002	0.000	0.006	0.151
SD	1.454	0.268	0.069	0.102	1.172	0.269	0.264	0.207	0.423	0.270	0.051	0.033	0.022	0.183	0.513	0.134
Min	0.880	0.000	0.000	0.000	0.000	0.000	0.001	0.000	0.004	0.020	0.000	0.000	0.000	0	0	0
Max	8.210	2.122	0.658	0.730	11.933	2.808	2.808	2.150	2.539	2.110	0.469	0.325	0.249	1.691	4.314	0.750
CV	0.44	1.82	1.68	1.34	1.13	2.42	1.81	3.24	0.94	0.73	3.75	2.04	3.10	4.09	3.60	0.83
SE	0.116	0.021	0.006	0.008	0.094	0.022	0.021	0.017	0.034	0.022	0.004	0.003	0.002	0.015	0.041	0.011
95% CI	(3.045, 3.501)	(0.105, 0.189)	(0.030, 0.052)	(0.060, 0.092)	(0.849, 1.217)	(0.069, 0.153)	(0.104, 0.187)	(0.031, 0.097)	(0.383, 0.516)	(0.325, 0.410)	(0.006, 0.022)	(0.011, 0.021)	(0.004, 0.011)	(0.016, 0.073)	(0.062, 0.223)	(0.140, 0.182)

SD: Standard deviation; Min: Minimum statistic; Max: Maximum statistic; CV: Co-efficient of variation; SE: Standard error; CI: Confidence Interval

The impact of the VOCs on different patients’ classification, such as cancer *vs*. control, cancer *vs*. benign, benign *vs*. control, (benign + cancer) *vs*. control, and (control + benign) *vs*. cancer, was studied through the Boot-SVM-RFE technique. The false discovery rates (FDR) for all the VOCs were computed through the Boot-SVM-RFE for different patients’ classifications and shown in Table 1. For cancer *vs*. control classification, 13 VOCs, including C_4_H_8_O and C_4_H_8_O_2,_ are found to be statistically significant when assessed through the FDR values (Fig 3, Table 2). Similarly, for cancer *vs*. benign, benign *vs*. control, and (benign + cancer) *vs*. control classifications, 15 VOCs were found to be statistically significant at the 1% level of significance (Fig 3, Table 1). Further, for (control + benign) *vs*. cancer classification, 13 VOCs were found to be statistically significant (Table 1). The rankings of the VOCs for five different patient classification problems are shown in Table 3. For instance, the VOCs such as C_4_H_8_O and C_4_H_8_O_2_ ranked 1 and 2 respectively for cancer *vs*. control classification (Table 3). These ranked VOCs can be used as biomarkers for cancer detection with respect to healthy controls. Similar interpretations about the ranking of the VOCs on other classifications can be made, as shown in Table 3. The empirical distributions of the significant VOCs are shown in Supplementary Document S4 in S1 File.

**Table 3:** Ranking of significant VOCs on different patients’ classification.

Ranks	Case I	Ranks	Case II	Ranks	Case III	Ranks	Case IV	Ranks	Case V
1	C_4_H_8_O	1	C_5_H_8_O	1	C_4_H_8_O_2_	1	C_4_H_8_O_2_	1	C_4_H_8_O_2_
2	C_4_H_8_O_2_	2	C_6_H_10_O_2_	2	C_13_H_22_O	2	C_13_H_22_O	2	C_6_H_10_O_2_
3	C_13_H_22_O	3	C_12_H_24_O	3	C_11_H_22_O	3	C_11_H_22_O	3	C_4_H_8_O
4	C_11_H_22_O	4	C_4_H_8_O_2_	4	C_2_H_4_O_2_	4	C_6_H_10_O_2_	4	C_13_H_22_O
5	C_2_H_4_O_2_	5	C_13_H_22_O	5	C_12_H_24_O	5	C_4_H_8_O	5	C_2_H_4_O_2_
6	C_6_H_10_O2	6	C_7_H_14_O	6	C_9_H_16_O_2_	6	C_7_H_14_O	6	C_11_H_22_O
7	C7H14O	7	C_13_H_26_O	7	C_7_H_14_O	7	C_9_H_16_O_2_	7	C_7_H_14_O
8	C9H16O2	8	C_6_H_12_O	8	C_5_H_8_O	8	C_2_H_4_O_2_	8	C_6_H_12_O
9	C6H12O	9	C_3_H_4_O	9	C_10_H_20_O	9	C_12_H_24_O	9	C_5_H_8_O
10	C13H26O	10	C_2_H_4_O_2_	10	C_3_H_4_O	10	C_5_H_8_O	10	C_13_H_26_O
11	C3H4O	11	C_4_H_8_O	11	C_13_H_26_O	11	C_6_H_12_O	11	C_9_H_16_O2
12	C8H16O	12	C_8_H_16_O	12	C_8_H_16_O	12	C_10_H_20_O	12	C_3_H_4_O
13	C5H8O	13	C_11_H_22_O	13	C_6_H_12_O	13	C_3_H_4_O	13	C_8_H_16_O
		14	C_9_H_16_O_2_	14	C_4_H_8_O	14	C_8_H_16_O		
		15	C_7_H_11_O	15	C_6_H_10_O_2_	15	C_13_H_26_O		

Case I: Cancer (156) vs. Control (193); Case II: Cancer (156) vs. Benign (65); Case III: Benign (65) vs. Control (193); Case IV: (Benign + Cancer) (221) vs. Control (193); Case V: (Control + Benign) vs. Cancer. Ranks of the VOCs based on the computed FDR values.

We also studied different combinations of the VOCs on classifying the patients into various classes, such as cancer *vs*. control, cancer *vs*. benign, benign *vs*. control, (benign + cancer) *vs*. control, and (benign + control) *vs*. cancer, through the SVM based classification model. Here, the classification accuracy was computed through a five-fold cross-validation technique for each classification problem. The cross-validations of the data were repeated 500 times by taking different combinations of VOCs (based on their ranks) for each classification problem. Then, the mean and standard error of the classification accuracies were computed over the 500 runs through a varying sliding window size technique [13, 16, 19]. The results are shown in Table 4. The results indicated that for cancer *vs*. control classification, the top three VOCs, including C_4_H_8_O, C_4_H_8_O_2_, and C_13_H_22_O, provided the reliable mean classification accuracy of 92.00% with a standard error of 0.03. Further, the highest mean classification accuracy was observed when the nine top-ranked VOCs (Table 3) were included in the data (Table 4). Similar interpretations can be made for other patients’ classification, such as cancer *vs*. benign, benign *vs*. control, (benign + cancer) *vs*. control, and (benign + control) *vs*. cancer. However, we observed consistently better results when the top-ranked nine VOCs (as given in Table 3) were considered in all the five classification problems.

**Table 4:** Classification metrics for different combinations of VOCs.

	Case I		Case II		Case III		Case IV		Case V	
#VOCs	Mean.CA	SE.CA	Mean.CA	SE.CA	Mean.CA	SE.CA	Mean.CA	SE.CA	Mean.CA	SE.CA
3	92.00	0.03	76.98	0.06	86.61	0.04	87.14	0.02	91.16	0.03
5	92.32	0.04	76.07	0.06	86.58	0.04	88.14	0.04	92.47	0.04
7	92.57	0.04	76.25	0.06	86.94	0.05	88.05	0.04	92.57	0.04
9	92.63	0.04	78.48	0.08	88.56	0.05	89.95	0.05	91.79	0.03
11	92.34	0.03	76.72	0.07	87.63	0.06	87.91	0.05	92.39	0.04
13	92.14	0.04	77.65	0.08	87.35	0.06	87.58	0.05	92.12	0.04
15	91.98	0.04	77.92	0.08	89.28	0.05	89.85	0.04	91.90	0.05

Case I: Cancer (156) vs. Control (193); Case II: Cancer (156) vs. Benign (65); Case III: Benign (65) vs. Control (193); Case IV: (Benign + Cancer) (221) vs. Control (193); Case V: (Control + Benign) vs. Cancer. Mean.CA: Mean Classification Accuracy; SE.CA: Standard Error in Classification Accuracy

**Fig 4:**
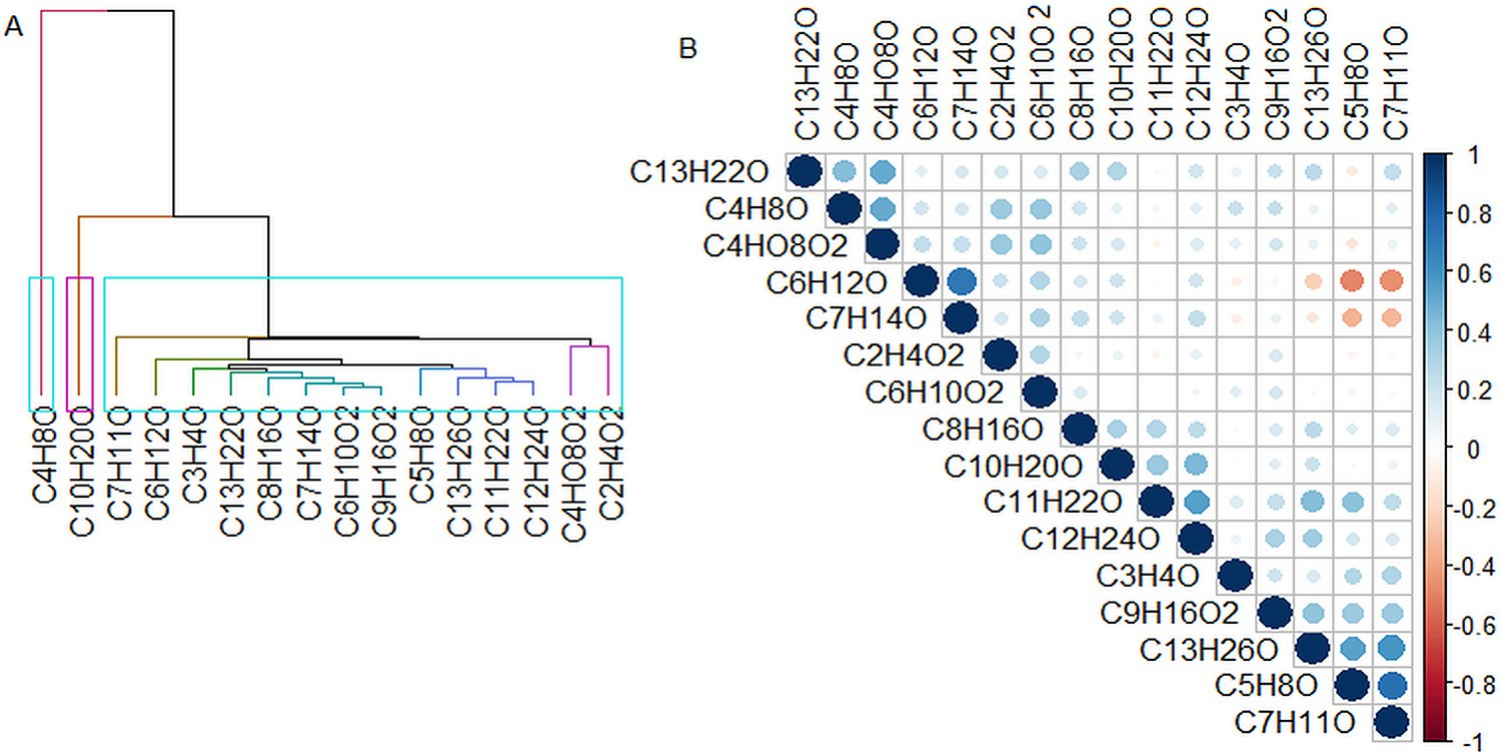
Similarity and correlation among the VOCs. (A) Dendrogram plot for the VOCs. A dendrogram is a diagram that shows the hierarchical relationship between the VOCs and is obtained through hierarchical clustering method. (B) Correlation plot for the VOCs. The correlation values with white spots represent non-significant correlation among the VOCs at 5% level of significance.

We also performed similarity analysis among the key detected VOCs across all the patients, and the results are shown in Fig 4. For instance, the distance-based similarity analysis of the 16 VOCs over all the patients is shown in Fig 4A. The results indicated that the VOCs, such as C_4_H_8_0 and C_10_H_20_O, are clustered separately (Fig 4A). These two VOCs have less similarity with others across all the patients (Fig 4A). The remaining VOCs are clustered together, which indicates their similarity over the samples irrespective of the cancer classes (Fig 4A). The correlation among the VOCs over all the patients is shown in Fig 4B. The correlation analysis indicated that the VOCs, *i*.*e*., C_5_H_8_O, C_13_H_26_O, and C_7_H_11_O, are negatively correlated with other VOCs across all the samples (Fig 4B). Further, the remaining VOCs are somewhat positively correlated with others. This analysis indicated a similarity in the molecular concentrations of the VOCs across the samples/patients observed through FT-ICR-MS technology.

This study demonstrates that few detected markers in exhaled breath samples can be used as marker signatures for distinguishing patients with lung cancer from patients with benign pulmonary nodules and healthy subjects (Supplementary Document S5 in S1 File). Here, the VOC markers for cancer detection are identified through the statistically sound Boot-SVM-RFE technique. Through this technique, a statistically meaningful measure, *i*.*e*., adjusted *p-value* or FDR, was computed after correcting the multiple hypothesis testing problems. Then the adjusted *p-value* or FDR was assigned to each VOC, and the significant VOCs were selected based on these computed values. This measure is easily interpretable by experimental biologists and lab users, as the values are well defined in [0, 1]. In other words, a lower *p-value* indicates a more informative VOC and *vice-versa*. The random resampling procedure, *i*.*e*., bootstrap method, used in the Boot-SVM-RFE can eliminate the spurious and arbitrary association among the VOCs while detecting marker signatures [13, 16, 19]. Further, the Boot-SVM-RFE is more robust and does not require any distributional assumptions of the VOC data to obtain the distribution of test statistic(s). After selecting a few VOC markers, we trained the machine learning (*i*.*e*., SVM) based classification models to establish their relevance in lung cancer patients’ classification. Our study found that instead of using all the VOCs, one can focus on few (*e*.*g*., 3, 5, or 7) marker VOCs to detect lung cancer patients more robustly and accurately. This approach will be less time-consuming and require lesser resources to detect lung cancer based on exhaled breath samples using FT-ICR-MS technology. Here, we have narrowed down our search to a few VOCs instead of focusing on all the VOCs present in breath samples, which in turn will save the time and cost of the experiments.

The major advantages of our experimental approach include: first, the microreactors are designed with thousands of micropillars to provide higher capture rates of carbonyl compounds (*e*.*g*., VOCs) in breath samples [10–12]. Second, chemo-selective capture of carbonyl compounds through amino-oxy reactions simplifies the spectrum of compounds to be quantitated. Both the steps are well established in the literature and can be further used to detect other diseases, including COVID19 using exhaled breath samples. Third, a statistically efficient technique, Boot-SVM-RFE [19], was used to detect the markers using the VOC molecular concentration data. Fourth, in-silico validation of the VOC signatures through training machine learning-based classification models. In other words, our experimental approach includes VOC molecular data generation through microreactors based FT-ICR-MS technology and statistical analysis of the data using efficient statistical and machine learning techniques.

## Conclusion

Early diagnosis of lung cancer is a key factor for increasing its survival rates among the patients. The analysis of carbonyl compounds present in exhaled breath of the patients is a promising non-invasive tool for the diagnosis of lung cancer at the early stage. In other words, the presence of metabolic carbonyl organic compounds in exhaled breath can play a vital role in the early detection of lung cancer patients, which will surely enhance the survival of the patients. Hence, the identification and characterization of the key metabolic VOCs using proper analytical approach and further using them in developing the classification models will play an important role in the quick and non-invasive detection of lung cancer. Therefore, in this study, we proposed an experimental approach to identify the key VOCs through the Boot-SVM-RFE technique and used these key VOCs to distinguish the patients with lung cancer from the benign pulmonary disease and healthy control classes.

Our analytical findings indicated that fewer VOCs can be used for lung cancer detection with sufficient classification accuracy. For instance, seven common key VOCs, including C_4_H_8_O_2_, C_13_H_22_O, C_11_H_22_O, C_2_H_4_O_2_, C_7_H_14_O, C_6_H_12_O, and C_5_H_8_O, can be successfully used for classification purposes under the five different settings. In this study, we used linear basis function in Boot-SVM-RFE technique, it will be interesting to study other non-linear basis functions or tree based models (e.g., random forests) to capture non-linear association among the VOCs. Further, our experimental and analytical approach of VOC quantitative analysis in breath samples may be extended to other diseases, including COVID19 detection. Besides, the analytical method used in this study can be applied to high-throughput gene expression studies, including RNA-sequencing and single-cell RNA-sequencing to select gene/bio-markers for the identification of cancer patients or cell types. Also, the reported experimental approach can be applied to other urine, saliva, and blood bio-assays based genetic studies to predict the phenotypes by identifying the organic compound based bio-signatures.

## Supporting information

S1 File Supplementary documents S1-S5. This file contains supporting documents from S1 to S5. Supplementary Document S1: SVM training for two class classification; Supplementary Document S2: Sliding Windows Size technique; Supplementary Document S3: List of VOC and their common names; Supplementary Document S4: Distribution of Key VOCs; Supplementary Document S5: Principal Component plots for visualizing patient classes.
(DOCX)

S1 Table Summary statistics of all the VOCs considered in this study. (XLSX)

S2 Table Statistical significance values computed through Boot-SVM-RFE for all the VOCs used in the study for all patient class combinations. (XLSX)
