# Supplementary material for: Multigroup prediction in lung cancer patients and comparative controls using signature of volatile organic compounds in breath samples
Source: PLoS One. 2022 Nov 30;17(11):e0277431. doi: 10.1371/journal.pone.0277431 (PMC9710764; doi:10.1371/journal.pone.0277431)
Supplement: S1 File — This file contains supporting documents from S1 to S5. Supplementary Document S1: SVM training for two class classification; Supplementary Document S2: Sliding Windows Size technique; Supplementary Document S3: List of VOC and their common names; Supplementary Document S4: Distribution of Key VOCs; Supplementary Document S5: Principal Component plots for visualizing patient classes. (DOCX) [file pone.0277431.s001.docx]

**Supplementary Documents**

**for**

**Multigroup Prediction in Lung Cancer Patients and Comparative Controls using Signature of Volatile Organic Compounds in Breath Samples**

Shesh N. Rai^1-6,*, ¶^, Samarendra Das^1, 2, 7-9, *,¶^, Jianmin Pan^1^, Dwijesh C. Mishra^9^, and Xiao-An Fu^10^

^1^Biostatistics and Bioinformatics Facility, Brown Cancer Center, University of Louisville, Louisville, KY 40202, USA

^2^School of Interdisciplinary and Graduate Studies, University of Louisville, Louisville, KY 40292, USA

^3^Hepatobiology and Toxicology Center, University of Louisville, Louisville, KY 40202, USA

^4^Department of Bioinformatics and Biostatistics, University of Louisville, Louisville, KY 40202, USA

^5^Biostatistics and Informatics Facility, Center for Integrative Environmental Research Sciences, University of Louisville, Louisville, KY, 40202, USA

^6^Christina Lee Brown Envirome Institute, University of Louisville, Louisville, KY 40202, USA

^7^ICAR-Directorate of Foot and Mouth Disease, Arugul, Bhubaneswar 752050, Odisha, India

^8^International Centre for Foot and Mouth Disease, Arugul, Bhubaneswar 752050, Odisha, India

^9^ICAR-Indian Agricultural Statistics Research Institute, PUSA, New Delhi 110012, India

^10^Department of Chemical Engineering, University of Louisville, Louisville, KY 40208, USA

**Authors’ email addresses:**

SNR: [shesh.rai@louisville.edu](mailto:shesh.rai@louisville.edu)

SD: [samarendra.das@louisville.edu](mailto:samarendra.das@louisville.edu)

JP: [jianmin.pan@louisville.edu](mailto:jianmin.pan@louisville.edu)

MB: [michael.bousamra@breath-dx.com](mailto:michael.bousamra@breath-dx.com)

DCM: [dwijesh.mishra@icar.gov.in](mailto:dwijesh.mishra@icar.gov.in)

XF: [xiaoan.fu@louisville.edu](mailto:xiaoan.fu@louisville.edu)

*The author whom all correspondence shall be addressed.

Email: [shesh.rai@louisville.edu](mailto:shesh.rai@louisville.edu), samarendra.das@louisville.edu

^¶^These authors contributed equally to this work and shall be considered as joint first authors.

**Supplementary Document S1: SVM training for two class classification**

**
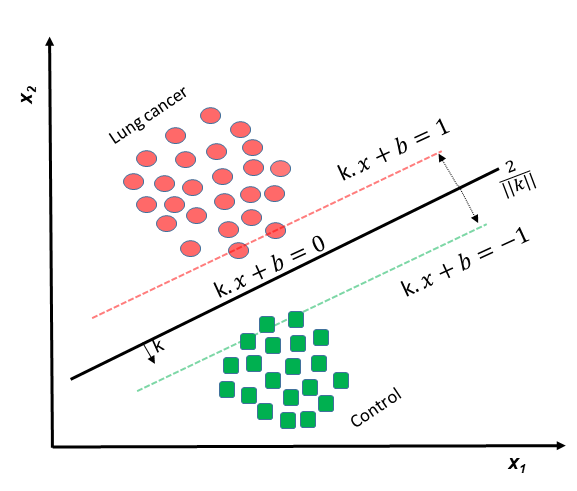
**

**Figure S1**. SVM training illustration with samples from two classes (i.e., control, cancer). $\boldsymbol{k}^{'}$ is N-dimensional vector of weights, $\boldsymbol{x}_{\boldsymbol{m}}$ is the N-dimensional vector of VOC observations for m^th^ patients, and b is the bias.

Here, it is assumed that the training data are linearly separable. Under this circumstance, we can select two hyperplanes that separate the two classes (e.g., lung cancer and control), so that the distance between them is maximum. In other words, we obtained the expressions of the weights for each through maximizing the distance between the hyperplanes. Here, the samples on/close to the hyperplanes, i.e., $\sum_{i=1}^{N} k_{i}x_{ip}\boldsymbol{+}b=1$and $\sum_{i=1}^{N} k_{i}x_{ip}\boldsymbol{+}b=-1$ are known as support vectors.

The following parameters considered for SVM training.

cost = 10, gamma = 0.001, cachesize=500, scale=F, type="C-classification", kernel="linear", probability=TRUE, cross=5

**Supplementary Document S2: Sliding Windows Size technique**

Sliding Windows Size technique is usually used in bioinformatics analysis to know the importance of positioning of the nucleotide bases in genomic intervals (referred as sliding windows). In this study, we used this technique to study the importance of rankings of VOCs (obtained from a feature selection method, e.g., SVM-RFE, Boot-SVM-RFE) through training a classification model. In other words, the sliding windows are VOC intervals that literally "slide" across the whole VOC list, almost always by some constant distance and classification accuracy is computed for each sliding window. Sliding windows can overlap or be mutually exclusive. The sliding window size technique is briefly illustrated in the following figure.


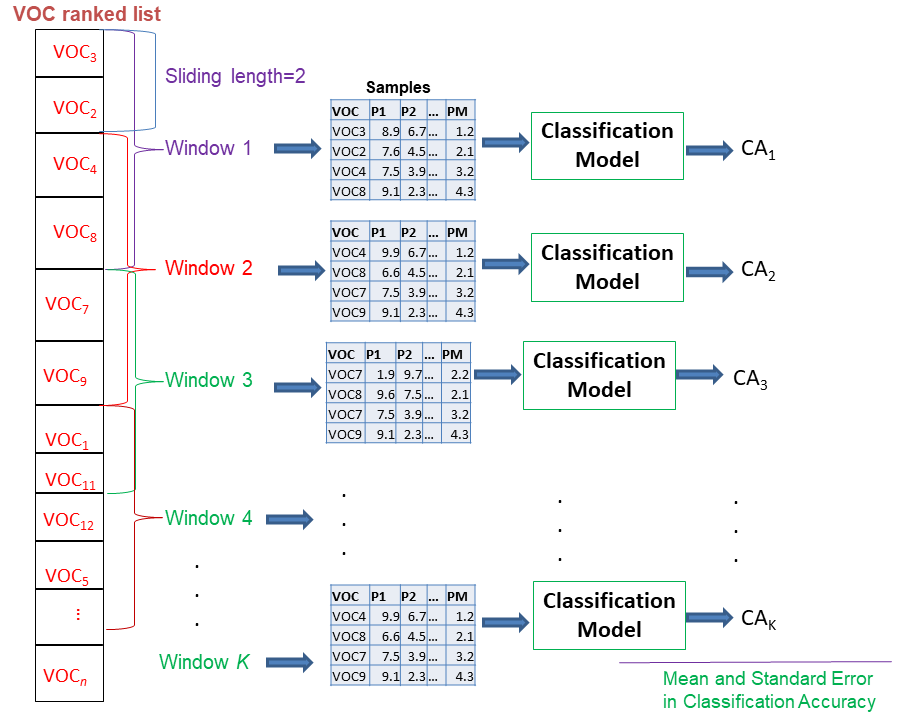


**Figure S2.** Illustration of the sliding windows size technique.

After computing the classification accuracies for each sliding windows, the overall impact of the rankings of the VOCs (obtained through the Boot-SVM-RFE) was observed using the criteria like mean and standard error of classification accuracies. The mean and standard error of classification accuracies are expressed as follows.

$\mu_{CA}=\frac{(\sum_{k=1}^{K} {CA}_{k})}{K}$

${SD}_{CA}=\sqrt{\frac{\sum_{k=1}^{K} {{(CA}_{k}-\mu_{CA})}^{2}}{(K-1)}}$

${SE}_{CA}=\frac{{SD}_{CA}}{\sqrt{K}}$

where, *K* is the total number of Windows. The total number of windows can be computed through following expression.

${K=(n-S)}/L$

where, *n* is the size of the VOC set, *S* is the size of the windows (*i.e.,* size refers to the number of ranked VOCs), and *L* is the sliding length.

In this study, we used the following sliding length, window size, and number of sliding windows to compute the measures.

| **Sl. No.** | **#VOCs (n)** | **Window size (S)** | **Sliding length (L)** | **Number of Sliding Windows (K)** |
| --- | --- | --- | --- | --- |
| 1 | 3 | 1 | 1 | 2 |
| 2 | 5 | 1 | 1 | 4 |
| 3 | 7 | 1 | 1 | 6 |
| 4 | 9 | 1 | 1 | 8 |
| 5 | 11 | 1 | 1 | 10 |
| 6 | 13 | 1 | 1 | 12 |
| 7 | 15 | 1 | 1 | 14 |

**Supplementary Document S3: List of VOC and their common names.**

| Formula | Common name of VOC |
| --- | --- |
| CH2O | Formaldehyde |
| C2H4O | Ethylene Oxide |
| C3H6O | Oxetane |
| C4H8O | Butyraldehyde |
| C5H10O | Pentanal |
| C6H12O | Hexanal |
| C7H14O | 2-Heptanone |
| C8H16O | Octanal |
| C9H18O | Nonanal |
| C10H20O | Decanal |
| C11H22O | Undecanal |
| C12H24O | _ |
| C13H26O | _ |
| C4H8O2 | Butyric acid |
| C2H4O2 | Acetic acid |
| C3H4O | Acrolein |
| C6H10O2 | 4-HHE |
| C9H16O2 | 4-HNE |
| C3H4O2 | MDA |
| C4H6O2 | Butanedione |
| C4H6O | Crotonaldehyde |
| C4H4O2 | Diketene |
| C5H8O | Cyclopentanone |
| C7H6O | Benzaldehyde |
| C7H11O | _ |
| C13H22O | Dicyclohexyl ketone |
| C15H10O | _ |

**Supplementary Document S4: Distribution of Key VOCs**

The distribution of key VOCs common to all cases (as mentioned in Figure 4) is shown below.


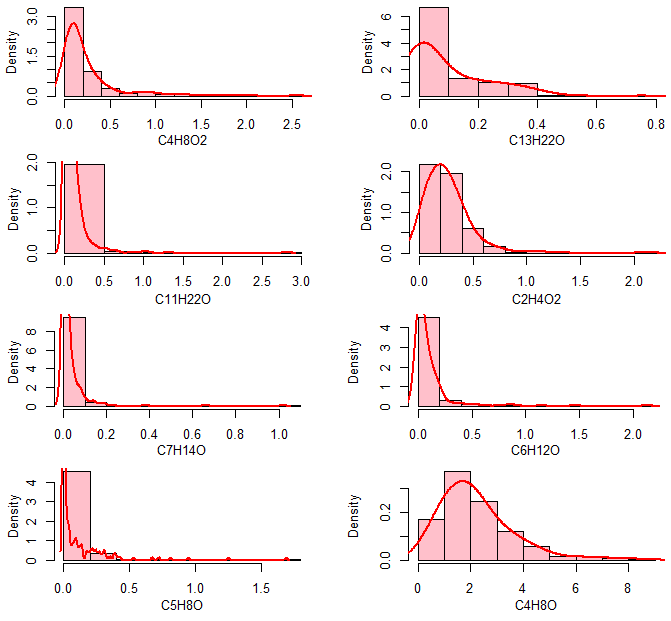


**Figure S3**. Distribution of key common VOCs across all the five different situations.


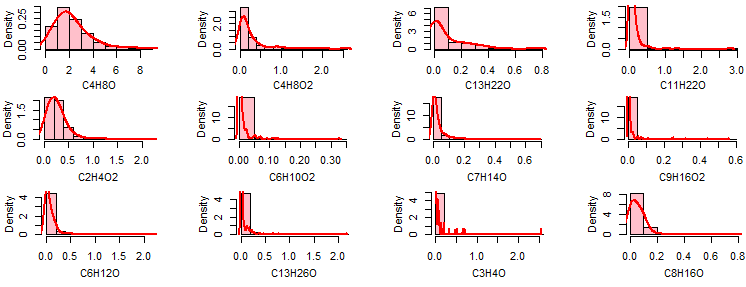


**Figure S4**: Distribution of the key VOCs identified through Boot-SVM-RFE technique in (Case I) Cancer *vs.* Control groups.


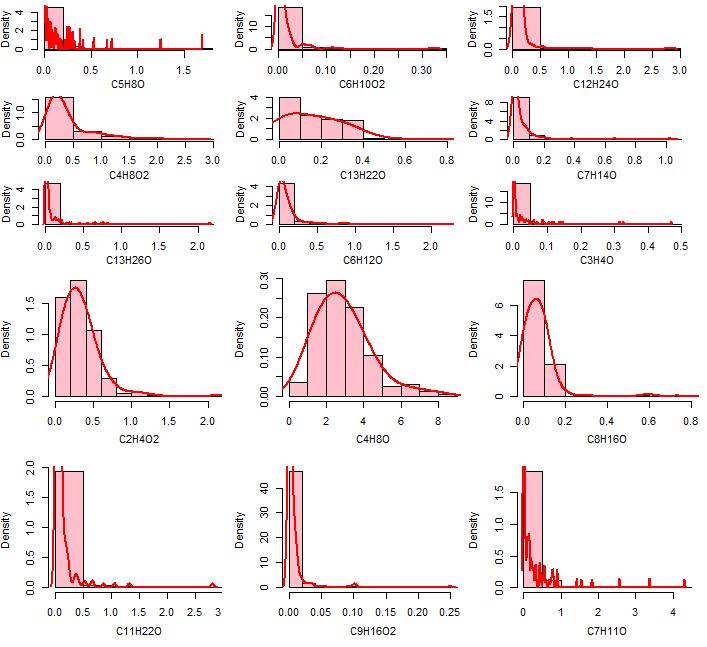


**Figure S5**: Distribution of the key VOCs identified through Boot-SVM-RFE technique in (Case II) Cancer *vs.* Benign patient classifications.


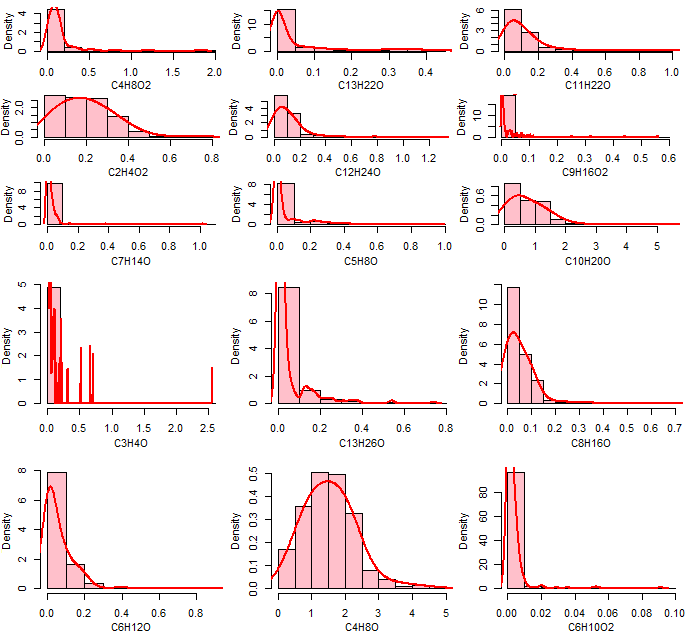


**Figure S6**: Distribution of the key VOCs identified through Boot-SVM-RFE technique in (Case III) Control *vs.* Benign patient classifications.


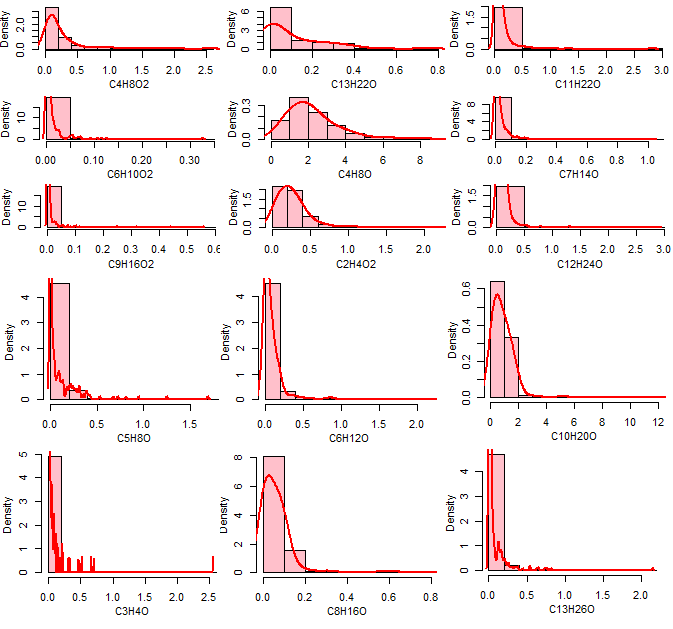


**Figure S7**: Distribution of the key VOCs identified through Boot-SVM-RFE technique in (Case IV) Control *vs.* (Benign + Cancer) patient classifications.


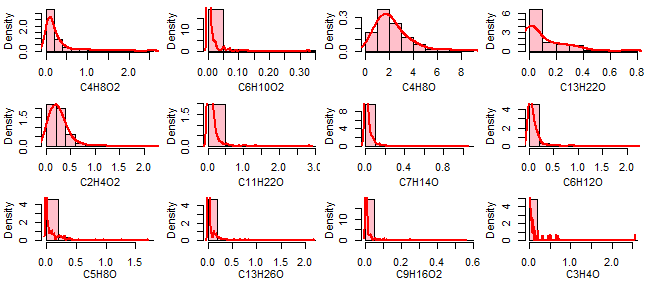


**Figure S8**: Distribution of the key VOCs identified through Boot-SVM-RFE technique in (Case V) (Control + Benign) *vs*. Cancer patient classifications.

**Supplementary Document S5: Principal Component plots for visualizing patient classes**


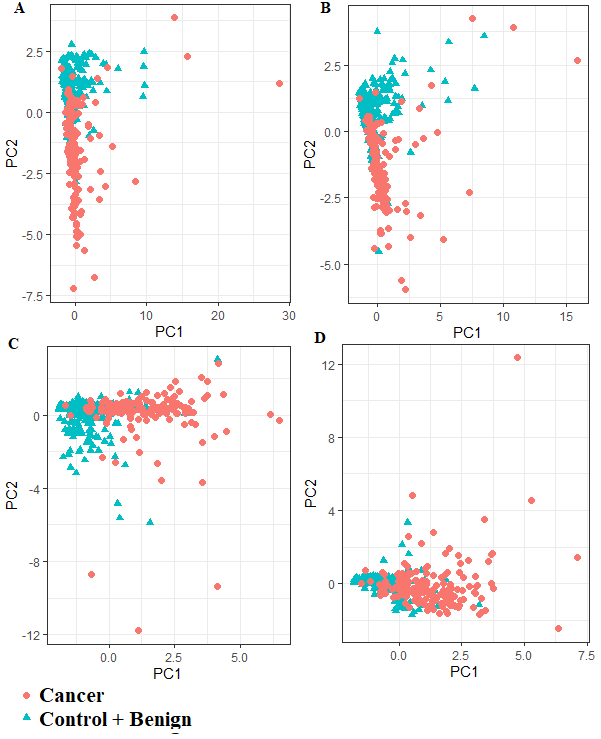


**Figure S9**: Principal Component Analysis (PCA) plot for visualization of patients’ classification into (Control + Benign) and Cancer classes. The patient classification is shown considering: (A) all the 28 VOCs; (B) 13 significant VOCs; (C) top-ranked 9 significant VOCs; (D) top-ranked 5 significant VOCs.


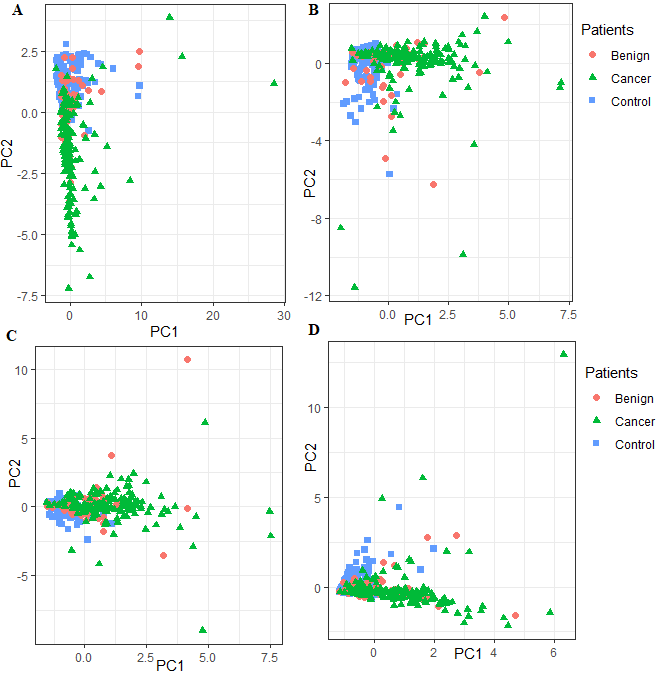


**Figure S10**: Principal Component Analysis (PCA) plot for visualization of patients’ classification into three classes, *i.e*., Control, Cancer, and Benign. The patient classification is shown considering: (A) all the 28 VOCs; (B) 7 significant VOCs; (C) top-ranked 5 significant VOCs; (D) top-ranked 3 significant VOCs.
